# Qualitative Histopathological Classification of Primary Bone Tumors Using Deep Learning: A Pilot Study

**DOI:** 10.3389/fonc.2021.735739

**Published:** 2021-10-06

**Authors:** Yuzhang Tao, Xiao Huang, Yiwen Tan, Hongwei Wang, Weiqian Jiang, Yu Chen, Chenglong Wang, Jing Luo, Zhi Liu, Kangrong Gao, Wu Yang, Minkang Guo, Boyu Tang, Aiguo Zhou, Mengli Yao, Tingmei Chen, Youde Cao, Chengsi Luo, Jian Zhang

**Affiliations:** ^1^ Department of Orthopaedics, The First Affiliated Hospital of Chongqing Medical University, Chongqing, China; ^2^ Department of Pathology, College of Basic Medicine, Chongqing Medical University, Chongqing, China; ^3^ Department of Pathology, The Second Affiliated Hospital of Chongqing Medical University, Chongqing, China; ^4^ Department of Pathology, Chongqing Hospital of Traditional Chinese Medicine, Chongqing, China; ^5^ Research and Development Department, Chongqing Defang Information Technology Co., Ltd, Chongqing, China; ^6^ Key Laboratory of Clinical Laboratory Diagnostics (Ministry of Education), College of Laboratory Medicine, Chongqing Medical University, Chongqing, China; ^7^ School of Life Science and Technology, University of Electronic Science and Technology of China, Chengdu, China

**Keywords:** primary bone tumors, deep learning, histopathological classification, convolutional neural network (CNN), diagnosis

## Abstract

**Background:**

Histopathological diagnosis of bone tumors is challenging for pathologists. We aim to classify bone tumors histopathologically in terms of aggressiveness using deep learning (DL) and compare performance with pathologists.

**Methods:**

A total of 427 pathological slides of bone tumors were produced and scanned as whole slide imaging (WSI). Tumor area of WSI was annotated by pathologists and cropped into 716,838 image patches of 256 × 256 pixels for training. After six DL models were trained and validated in patch level, performance was evaluated on testing dataset for binary classification (benign *vs*. non-benign) and ternary classification (benign *vs*. intermediate *vs*. malignant) in patch-level and slide-level prediction. The performance of four pathologists with different experiences was compared to the best-performing models. The gradient-weighted class activation mapping was used to visualize patch’s important area.

**Results:**

VGG-16 and Inception V3 performed better than other models in patch-level binary and ternary classification. For slide-level prediction, VGG-16 and Inception V3 had area under curve of 0.962 and 0.971 for binary classification and Cohen’s kappa score (CKS) of 0.731 and 0.802 for ternary classification. The senior pathologist had CKS of 0.685 comparable to both models (*p* = 0.688 and *p* = 0.287) while attending and junior pathologists showed lower CKS than the best model (each *p* < 0.05). Visualization showed that the DL model depended on pathological features to make predictions.

**Conclusion:**

DL can effectively classify bone tumors histopathologically in terms of aggressiveness with performance similar to senior pathologists. Our results are promising and would help expedite the future application of DL-assisted histopathological diagnosis for bone tumors.

## 1 Introduction

Primary bone tumors are a variety of neoplasms formed from the bone tissue (1). Although the incidence is relatively low, primary bones and joints’ malignancy is ranked the third and fourth leading cause of death for males and females under 20 years of age in the United States (2). The biological behavior of bone tumors varies greatly among different classes (3). However, their clinical management is mainly determined by the extent of the tumor’s aggressiveness, which is usually graded as benign, intermediate, and malignant (4). While the bone tumor’s clinical characteristics and radiological information may help physicians reach an initial diagnosis, histopathological assessment of biopsy tissue remains decisive in determining the bone tumor’s biological nature and confirming its aggressiveness (5). Therefore, an accurate and reliable histopathological differentiation is imperative to ensure a satisfactory patient outcome.

Unlike tumors of epithelial origin that are more prevalent, pathologists’ experience in diagnosing bone tumors usually lacks due to the relatively low incidence and various histological morphology. Additionally, some bone tumors of different kinds may share similar histologic morphology because of mesenchymal origin, thus introducing confounding factors in classification. Moreover, the pathologist’s prediction of bone tumor’s histopathological classification, which is prone to subjectivity, could not be adequately quantified for the moment.

Considering the drawbacks of traditional histopathological analysis mentioned above, diagnostic approaches based on artificial intelligence gradually come into existence, along with the accelerated development of computational power and deep learning (DL) (6). The convolutional neural network (CNN), a network composed of deep layers, can be trained to extract specific features from an image dataset to output a quantitative probability and build a classifier (7). In addition, the emergence of whole slide imaging (WSI) enables slides digitalized as macro data without information loss (8), which is suitable for neural networks to process and learn. Utilizing WSI over the last few years, the CNN has been verified efficient in the histopathological classification of numerous tumors of epithelial origin, such as breast cancer (9), lung cancer (10), gastric cancer (11), prostate cancer (12), and nasopharyngeal cancer (13). In comparison to tumors of epithelial origin, bone tumors are mostly of mesenchymal origin, showing remarkably different and diverse microscopic morphology. However, there lacks relevant evidence regarding the performance of DL-based histopathological classification for bone tumors so far.

Accurate DL-assisted differentiation of primary bone tumors microscopically and qualitatively as benign, intermediate, and malignant would not only compensate for the limited experience and biased interpretation of physicians, but also provide a quantitative approach to assess the biological nature of bone tumors, potentially leading to a better treatment decision. In this study, we evaluate the feasibility of using DL in qualitative histopathological differentiation of primary bone tumors and compare the performance of the best model with pathologists of different levels of experience.

## 2 Materials and Methods

### 2.1 Specimen Information

According to the 1964 Helsinki declaration and its later amendments, this study was approved by the ethics committee of the First Affiliated Hospital of Chongqing Medical University (No. 2020-287). After ensuring that informed consents were obtained from relevant patients, all specimens of primary bone tumor resected in the hospital between July 2014 and October 2020 were retrieved from the Department of Pathology, Chongqing Medical University. Based on the histopathological, clinical, and radiological information, the collected samples’ diagnoses were confirmed by at least one senior pathologist in accordance with the 2013 World Health Organization (WHO) classification (4). A total of 458 specimens were finally determined and classified into three groups, in which 206 were benign, 96 were intermediate, 156 were malignant.

### 2.2 Data Preparation

#### 2.2.1 Section and Staining

The collected paraffin-embedded specimens were sectioned and stained under a standardized protocol, producing one corresponding hematoxylin and eosin (H&E) slide for each specimen. All slides were de-identified and only labeled with diagnosis. The quality control of all slides was done by a senior pathologist, and 31 slides (8 benign, 10 intermediate, and 13 malignant cases) were excluded from the study. The remaining 427 slides were finally chosen for scanning. **Supplementary Table S1** shows the detailed number of cases with definitive diagnoses in each group. The average age of the included cases was 38.06 years (from 7 to 89 years), while males and females accounted for 53.62% and 46.37%, respectively.

#### 2.2.2 WSI Scanning and Storage

**Figure 1:**
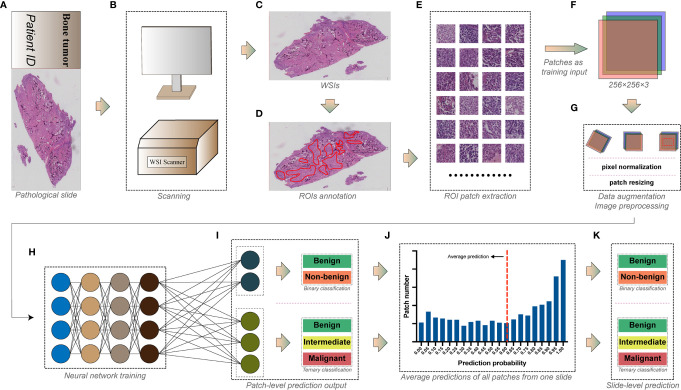
The workflow for deep learning training. **(A)** The pathological slide prepared from bone tumor specimens. **(B)** Whole slide imaging (WSI) scanning. **(C)** Whole slide images with ultra-high resolution. **(D)** Tumor areas annotated by pathologists as regions of interest (ROIs). **(E)** Regions of interest were exported as numerous square image patches of the same size. **(F)** Three-channel RGB image patches of 256 × 256 pixels were used as training input. **(G)** Data augmentation and image preprocessing before training. **(H)** Image patches in the training dataset were fed into the convolutional neural network for training. **(I)** Models were trained for the patch-level binary and ternary classification. **(J)** Predictions of all patches from one slide were averaged to obtain **(K)** the slide-level binary and ternary prediction.

The selected slides were scanned using a digital slide scanner (Chongqing Defang Information Technology Co., Ltd, Chongqing, China) to produce ultra-high-resolution whole slide images at the default 40× objective magnification (**Figures 1A–C**), which then were stored as svs format. The average memory size of all WSIs was 5.76 GB, and the width and height of WSIs were at least 149,520 and 150,420 pixels.

#### 2.2.3 Annotation

WSIs were analyzed by pathologists using Qupath (14) (version 0.2.3, Queens University). Areas constituted of tumor-related cells and structures were considered as viable tumor areas, while other non-specific normal connective tissues and white space were regarded as non-tumor areas. Two junior pathologists examined all WSIs under 1× to 40× objective magnification before determining and annotating viable tumor areas as regions of interest (ROIs) using Qupath built-in annotation tools (**Figure 1D**). WSIs were subsequently rechecked by another senior pathologist to ensure the accuracy of annotation.

#### 2.2.4 Dataset Allocation

All WSIs under each group were randomly split into training, validation, and testing datasets in a proportion of 70:15:15. Slide dataset information for each group is shown in **Supplementary Table S2**.

#### 2.2.5 Image Patch Extraction

WSIs are images with more than hundreds of millions of pixels (8), which are too huge to be used as input in training DL models. Moreover, the discriminative information of histopathology is usually retained at the cellular level (15). Therefore, ROIs of WSIs are usually cropped into plenty of image patches with fixed dimensions (typically from 32 × 32 to 10,000 × 10,000 pixels, where 256 × 256 is the most widely used) as input, making the training possible and efficient (16). As a result, we used the Qupath script editor to continuously crop the annotated viable tumor areas into square image patches of 256 × 256 pixels without overlapping (**Figure 1E**). In this study, we used a down-sampling factor of four when cropping the ROIs because the image patch of 256 × 256 pixels generated from the original 40× scanning magnification was insufficient to include a satisfactory tumor area. Image patches with background constituting more than 50% of their areas were abandoned. The cropped patches share the same group label as the slides from which they were generated. A total of 716,838 patches were finally generated, and detailed information of image patches for each group is shown in **Supplementary Table S3**.

### 2.3 Network Training and Performance Evaluation

Several widely tested convolutional neural network architectures, including AlexNet (17), VGG-16 (18), Inception V3 (19), DenseNet-121 (20), ResNet-50 (21), and MnasNet (22) were chosen for training the patch-level classification. All image patches extracted were saved in 8-bit JPEG format (**Figure 1F**). We performed the data augmentation and preprocessing by random rotation, random horizontal flip, and normalization of the original image (**Figure 1G**). The angle of random rotation ranged from −45° to 45°. The probability of images being flipped was 0.5. Pixel values of three-channel images were normalized by scaling their values into the range from zero to one, then subtracting [0.485, 0.456, 0.406] and dividing by [0.229, 0.224, 0.225] channel-wise. Random resized cropping was used such that a crop of random size (0.08 to 1.0) of the original size and a random aspect ratio (of 3/4 to 4/3) of the original aspect ratio was made, finally resizing the image to a given size (224 × 224 or 299 × 299, according to the model’s pre-trained dataset, shown in **Supplementary Figures S1–S6**) as training input.

All models were pre-trained on the ImageNet dataset to initialize kernel weights. Stochastic gradient descent (SGD) with a categorical cross-entropy loss was implemented to update the model’s weights, accompanied by a cyclic learning rate (23) (cLR) oscillating between 10^-4^ and 10^-6^ every quarter epoch. The batch size of 64 was set for training. Models were trained on patch level (**Figures 1H, I**) for binary classification (benign *vs*. non-benign) and a ternary classification (benign *vs*. intermediate *vs*. malignant). The architecture and specific hyper-parameters of each model are shown in **Supplementary Figures S1–S6**.

The model’s generalizability for each epoch during training was evaluated with validation dataset using loss and accuracy for binary classification or using loss, accuracy, and the Cohen’s kappa score (CKS) for ternary classification. All models were trained for 30 epochs, and parameters of the epoch with the highest validation accuracy (binary task) or CKS (ternary task) were used to predict the classification of the testing dataset. We compared the patch-level diagnostic metrics on the testing dataset between different models and determined the best architecture, which was then used to predict the slide-level classification.

The model’s predictions of all image patches generated from one slide WSI were averaged to produce a slide-level prediction (**Figures 1J, K**). Then, the true label of the slide was used to assess the model’s slide-level classification performance on the testing dataset.

Metrics of performance for binary classification included accuracy, sensitivity, specificity, positive predictive value (PPV), negative predictive value (NPV), F1-score, the receiver operating characteristic (ROC) curve, and the area under the curve (AUC), whereas accuracy, the Cohen’s kappa score, precision, recall, and F1-score were used to evaluate the model’s ternary classification performance.

### 2.4 Experiment Setup

Our DL experiments were performed on a server with 4× NVIDIA GeForce RTX 2080 Ti graphics processing units (11 GB of memory for each). We developed the relevant DL algorithms with Python 3.6 and PyTorch 1.7.1 on an Ubuntu platform.

### 2.5 Evaluation of Pathologist’s Performance

All slides in the testing dataset were read by one senior pathologist (pathologist #1, with more than 25 years of experience), two attending pathologists (pathologist #2 and #3, with more than 10 years of experience), and one resident pathologist (pathologist #4, with less than 5 years of experience) without knowing any slide’s information beforehand. Then, they labeled each slide as benign, intermediate, or malignant according to their own interpretations. Their predictions of all slides were recorded and compared with the slides’ corresponding ground-truth labels to calculate the pathologist’s diagnostic performance. Metrics used in the model’s slide-level performance evaluation were analyzed for pathologists and finally compared between model and human.

### 2.6 Model Visualization and Case Review

Gradient-weighted class activation mapping (Grad-CAM) is an approach that uses the gradients flowing into the last convolutional layer to create a map localizing and highlighting the important regions relevant to model prediction in an image (24). Therefore, we used Grad-CAM to visualize the important regions associated with discriminative histopathological features that the DL model relies on, thus revealing the underlying mechanism of the model’s prediction. In the slide-level classification, we identified the slide cases that the model, or pathologist, or both wrongly classified. Then, the senior pathologist was asked to determine the potential causes of such misclassifications by reviewing the representative image patches of the corresponding slide, along with the model visualization.

### 2.7 Statistical Analysis

Data used in this study were analyzed with SPSS software (version 26.0; IBM, Chicago, IL) and SAS 9.4 (SAS Institute, Cary, NC, USA). The metrics of performance for slide-level binary classification between models and pathologists were compared using McNemar’s test. The 95% confidence intervals (CIs) of AUCs were calculated and compared between groups using the Delong methods (25), and the 95% CIs of the Cohen’s kappa scores were acquired by the bootstrap method (26) with 10,000 replications and compared between the model and the pathologist using the permutation test with 10,000 iterations. The AUC in different ranges represented the following predictive performance: poor (0.5 ≤ AUC < 0.7), fair (0.7 ≤ AUC < 0.8), good (0.8 ≤ AUC < 0.9), and excellent (0.9 ≤ AUC). We characterize the Cohen’s kappa score of 0–0.20, 0.21–0.41, 0.41–0.60, 0.61–0.80, and 0.81–1 as slight, fair, moderate, substantial, excellent agreement with the ground truth label, respectively. A *p*-value of less than 0.05 was considered statistically significant.

## 3 Result

### 3.1 Patch-Level Performance of Models

#### 3.1.1 Binary Classification

**Figure 2:**
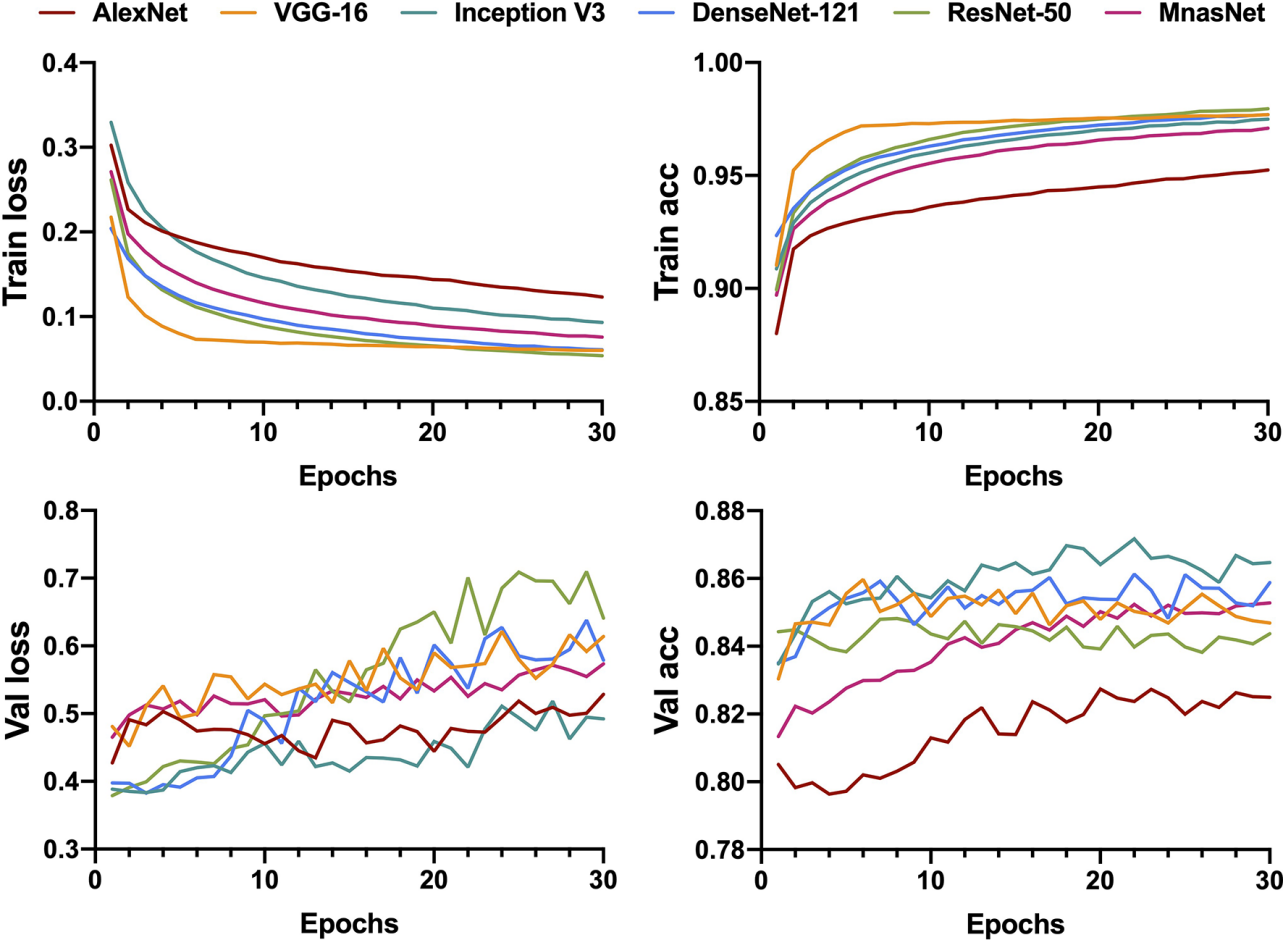
Learning curves for patch-level binary classification of six models showing the loss and accuracy in training and validation. Train, training; Val, validation; acc, accuracy.

All generated patches from the training dataset were fed into six pre-trained CNN models to build a binary classifier (benign *vs*. non-benign). The learning curves for 30 epochs of all models are shown in **Figure 2**. The validation loss of most models reached the lowest level in the first 15 epochs before rising slowly afterwards, indicating that the models gained the high level of generalizability in the initial training process.

**Figure 3:**
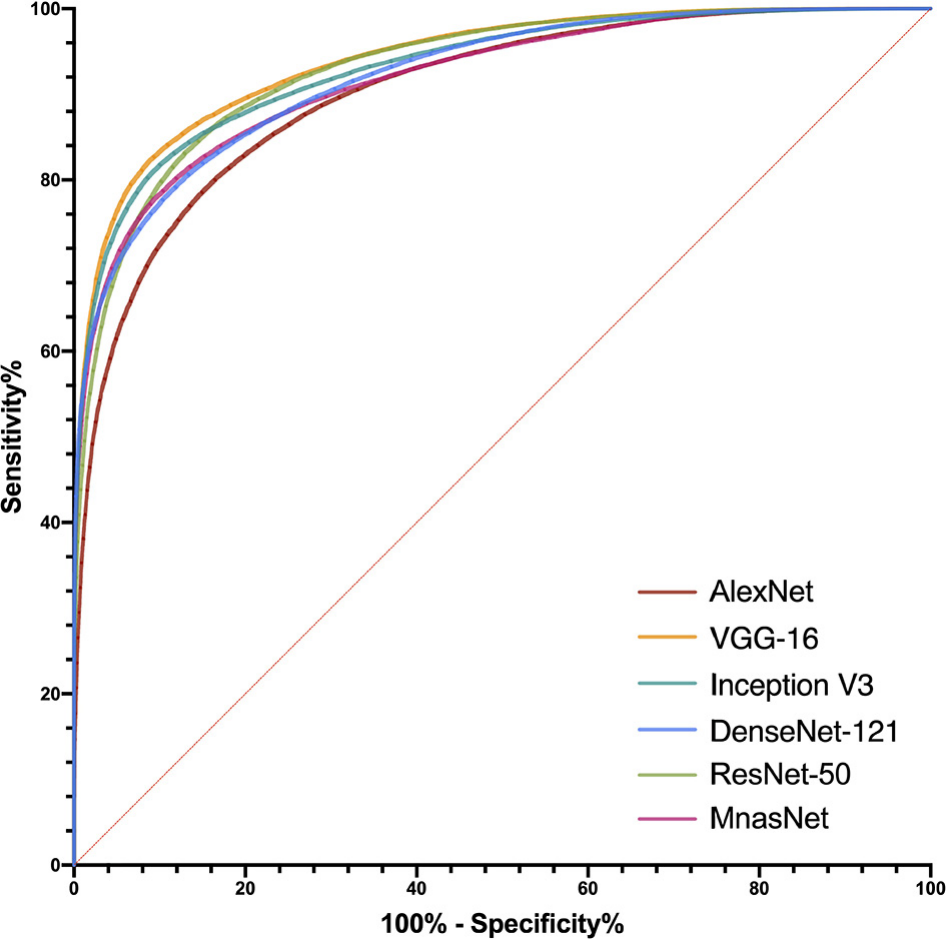
Receiver operating characteristic curves in the patch-level binary classification of each model.

After determining models’ best-performing parameters using validation accuracy, we assessed the performance of models’ patch-level binary classification on the testing dataset. **Figure 3** depicts the ROC curves for each model, where the VGG-16 showed the best predictive value with an AUC of 0.940 (95% CI, 0.939–0.941), while the AlexNet had the smallest AUC of 0.902 (95% CI, 0.939–0.941) among six models. For other diagnostic metrics, the VGG-16 also had the highest accuracy (85.96%), sensitivity (83.66%), NPV (77.78%), and F1-score (87.91%) compared with other network architectures, whereas the Inception V3 showed the greatest specificity (91.34%) and PPV (93.56%). The detailed information of performance metrics for patch-level binary classification is demonstrated in **Table 1**. Therefore, we chose the VGG-16 and the Inception V3 for slide-level binary prediction.

**Table 1:** Performance of patch-level binary classification on testing dataset.

	Accuracy (%)	Sensitivity (%)	Specificity (%)	PPV (%)	NPV (%)	F1-score (%)	AUC (95% CI)
**AlexNet**	81.13	78.77	84.83	89.05	71.85	83.59	0.902 (0.900, 0.904)
**VGG-16**	**85.96**	**83.66**	89.58	92.63	**77.78**	**87.91**	**0.940** (0.939, 0.941)
**Inception V3**	84.62	80.32	**91.34**	**93.56**	74.78	86.44	0.930 (0.929, 0.932)
**DenseNet-121**	83.02	81.02	86.16	90.16	74.35	85.34	0.922 (0.920, 0.923)
**ResNet-50**	84.73	83.58	86.54	90.67	77.09	86.98	0.930 (0.929, 0.932)
**MnasNet**	83.32	80.68	87.46	90.97	74.31	85.52	0.918 (0.916, 0.919)

Metric with the greatest value among different models is bolded. PPV, positive predictive value; NPV, negative predictive value; AUC, area under the curve; CI, confidence interval.

#### 3.1.2 Ternary Classification

**Figure 4:**
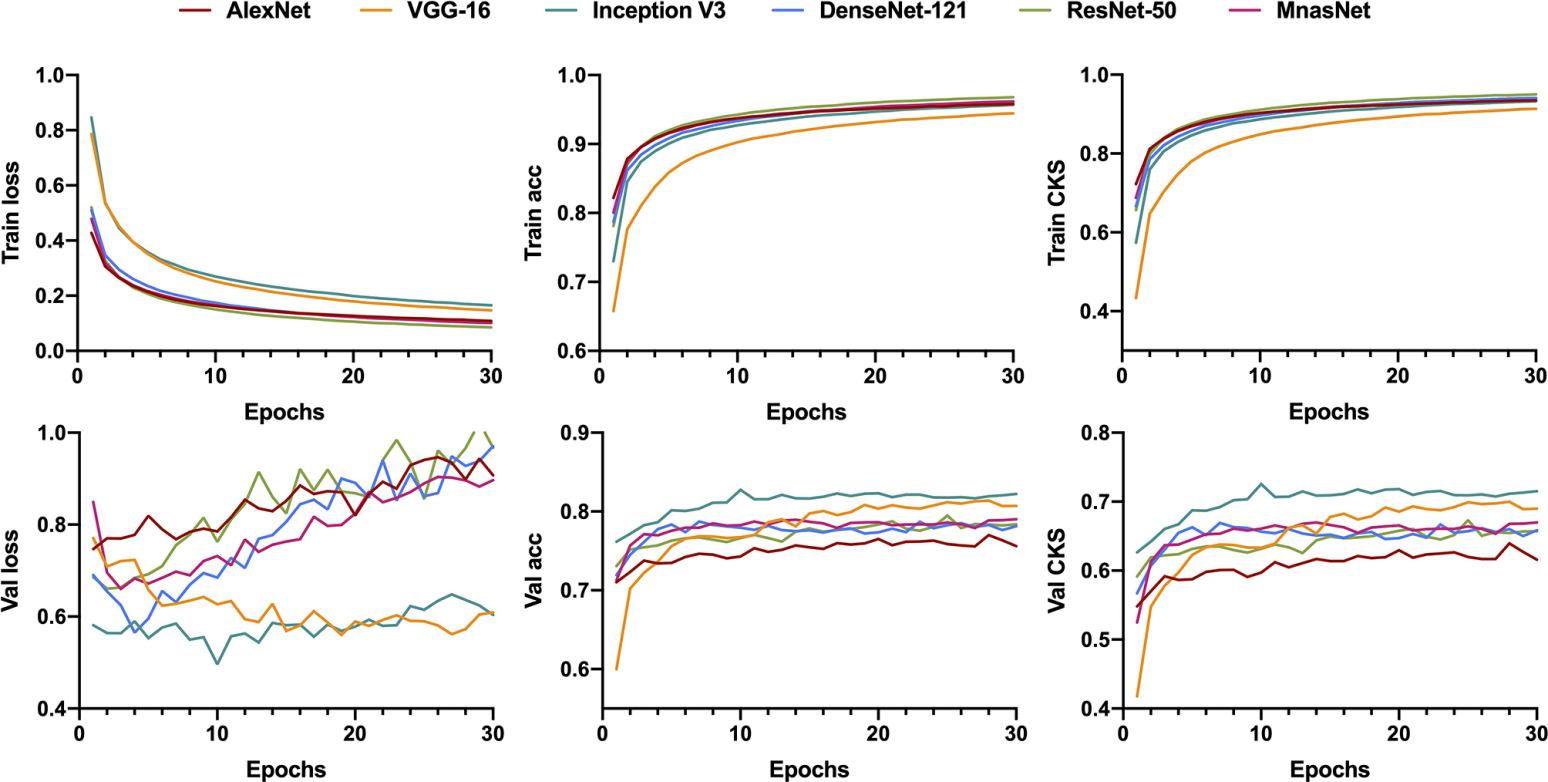
Learning curves for patch-level ternary classification of six models showing the loss, accuracy, and the Cohen’s kappa score in training and validation. Train, training; Val, validation; acc, accuracy; CKS, the Cohen’s kappa score.

Similar to the training for binary classification, six models were fed with patches that were labeled as benign, or intermediate, or malignant to train a ternary classifier. However, we utilized the CKS, rather than accuracy, to decide the best parameters in the training process because of the relative imbalance of the patch number between each class in ternary classification. **Figure 4** illustrates the learning curves for 30 epochs of each model. As the epoch increased, the validation loss of VGG-16 and Inception V3 was fairly stable at a low level compared with the other four models, showing less chance of overfitting for these two models.

For the model’s performance in patch-level ternary classification on testing dataset, the VGG-16 triumphed over others on accuracy (74.78%), CKS (0.601, 95% CI 0.597–0.605), weighted average recall (0.75), and weighted average F1-score (0.75), while sharing the highest weighted average precision (0.79) with the Inception V3. **Table 2** summarizes the performance metrics of six models, and the detailed classification report for each class is shown in **Supplementary Table S4**. As a result, the VGG-16 and the Inception V3 were finally selected for slide-level ternary prediction.

**Table 2:** Performance of patch-level ternary classification on testing dataset.

	Accuracy (%)	Cohen’s kappa score (95% CI)	WA precision	WA recall	WAF1-score
**AlexNet**	68.62	0.505 (0.501, 0.509)	0.73	0.69	0.69
**VGG-16**	**74.78**	**0.601** (0.597, 0.605)	**0.79**	**0.75**	**0.75**
**Inception V3**	74.17	0.591 (0.587, 0.595)	**0.79**	0.74	0.74
**DenseNet-121**	72.48	0.570 (0.566, 0.574)	0.78	0.72	0.73
**ResNet-50**	70.02	0.527 (0.523, 0.531)	0.74	0.70	0.70
**MnasNet**	73.39	0.575 (0.571, 0.579)	0.76	0.73	0.73

Metric with the greatest value among different models is bolded. CI, confidence interval; WA, weighted average.

### 3.2 Slide-Level Performance of Models and Pathologists

#### 3.2.1 Binary Classification

**Figure 5:**
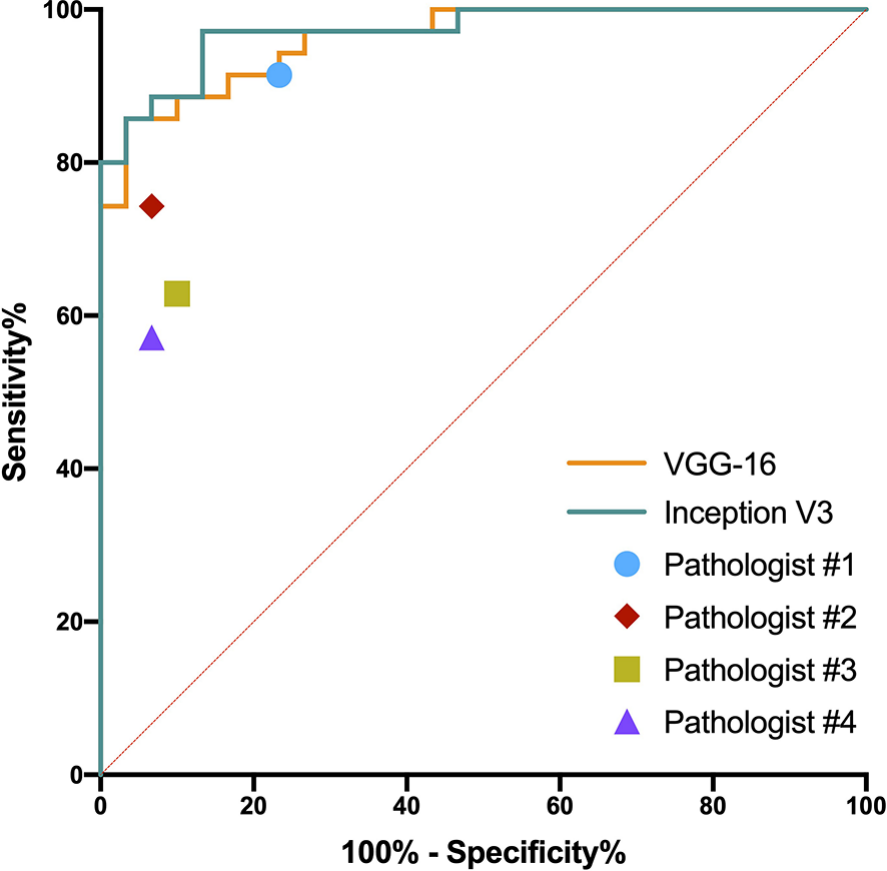
Receiver operating characteristic curves in the slide-level binary classification of VGG-16 and Inception V3, along with results of pathologists’ assessments.

The predictive probabilities of all patches generated from one slide were averaged to obtain the model’s slide-level prediction. For the differentiation of benign from non-benign bone tumors on the testing dataset, the VGG-16 and the Inception V3 both showed excellent predictive capability on slide-level with the AUC of 0.962 (95% CI, 0.882–0.994) and 0.971 (95% CI, 0.897–0.997), respectively. In addition, there was no statistically significant difference between the AUCs of both models (*p* = 0.304). The ROC curves for slide-level binary classification of models are demonstrated in **Figure 5**, along with the results of pathologists’ assessments. **Table 3** summarizes the detailed performance metrics for models and pathologists. Among models and pathologists, the VGG-16 had the highest accuracy (90.77%) and F1-score (90.91%), and the Inception V3 showed the greatest specificity (100.00%) and PPV (100.00%). Senior pathologist #1 had the best accuracy (84.62%) among pathologists, while owning better sensitivity (91.43%) and NPV (88.46%) compared with models. However, the heterogeneity of predictive performance among pathologists was significant that their sensitivities and specificity ranged from 57.14% and 76.67% to 91.43% and 93.33%, respectively. The *p*-values for comparison of accuracy, sensitivity, and specificity between VGG-16 and pathologists are shown in **Table 3**.

**Table 3:** Performance of slide-level binary classification on testing dataset.

	Accuracy (%)	Sensitivity (%)	Specificity (%)	PPV (%)	NPV (%)	F1-score (%)	AUC (95% CI)
**VGG-16**	**90.77**	85.71	96.67	96.77	85.29	**90.91**	0.962 (0.882, 0.994)
**Inception V3**	87.69	77.14	**100.00**	**100.00**	78.95	87.10	**0.971** (0.897, 0.997)
**Pathologist #1**	84.62^a1^	**91.43** ^b1^	76.67^c1^	82.05	**88.46**	86.49	–
**Pathologist #2**	83.08^a2^	74.29^b2^	93.33^c2^	92.86	75.68	82.54	–
**Pathologist #3**	75.38^a3^	62.86^b3^	90.00^c3^	88.00	67.50	73.33	–
**Pathologist #4**	73.85^a4^	57.14^b4^	93.33^c4^	90.91	65.12	70.18	–

Metric with the greatest value among different groups is bolded. ^a1-4,b1-4,c1-4^indicate the p-values compared with the VGG-16 in accuracy, sensitivity, and specificity, respectively. a1, p = 0.317; a2, p = 0.096; a3, p = 0.012; a4, p = 0.008; b1, p = 0.480; b2, p = 0.103; b3, p = 0.021; b4, p = 0.008; c1, p = 0.034; c2, p = 0.564; c3, p = 0.317; c4, p = 0.564. PPV, positive predictive value; NPV, negative predictive value; AUC, area under the curve; CI, confidence interval.

#### 3.2.2 Ternary Classification

Slide-level ternary classification performances of models and pathologists are outlined in **Table 4**. The Inception V3 had the greatest value in each metric. Both the VGG-16 and the Inception V3 showed substantial predictive value with the CKS of 0.731 (95% CI, 0.573–0.860) and 0.802 (95% CI 0.662–0.920), whereas pathologists of all levels had the CKSs of less than 0.7. However, after pairwise comparison of CKS, we found that there were no significant differences between the VGG-16 and the Inception V3 (*p* = 0.182), the VGG-16 and pathologist #1 (*p* = 0.689), and the Inception V3 and pathologist #1 (*p* = 0.288). The CKSs of pathologists #2–4 were significantly lower than the Inception V3 (see **Table 4** for the detailed *p*-values). The detailed classification report for each class is shown in **Supplementary Table S5**.

**Table 4:** Performance of slide-level ternary classification on testing dataset.

	Accuracy (%)	Cohen’s kappa score (95% CI)	WA precision	WA recall	WAF1-score
**VGG-16**	83.10	0.732 (0.574, 0.867)	0.83	0.83	0.82
**Inception V3**	**87.70**	**0.803** (0.664, 0.922)	**0.90**	**0.88**	**0.87**
**Pathologist #1**	80.00	0.686 (0.526, 0.829) ^a1,b1^	0.81	0.80	0.80
**Pathologist #2**	72.31	0.543 (0.376, 0.703) ^a2,b2^	0.70	0.72	0.70
**Pathologist #3**	69.23	0.490 (0.307, 0.664) ^a3,b4^	0.70	0.69	0.68
**Pathologist #4**	70.77	0.507 (0.335, 0.679) ^a4,b4^	0.74	0.71	0.69

Metric with the greatest value among different groups is bolded. ^a1-4^indicates the p-value compared with the VGG-16 and ^b1-4^indicates the p-value compared with the Inception V3. a1, p = 0.689; a2, p = 0.060; a3, p = 0.036; a4, p = 0.048; b1, p = 0.288; b2, p = 0.004; b3, p = 0.002; b4, p = 0.003. CI, confidence interval; WA, weighted average.

### 3.3 Model Visualization

We located the slides that were correctly classified by both models (VGG-16 and Inception V3) and the senior pathologist #1 in binary and ternary classification, then chose the representative patches of selected slides for Grad-CAM visualization. The model VGG-16 was used for visualization because it showed the best binary and ternary patch-level predictive performances.

**Figure 6:**
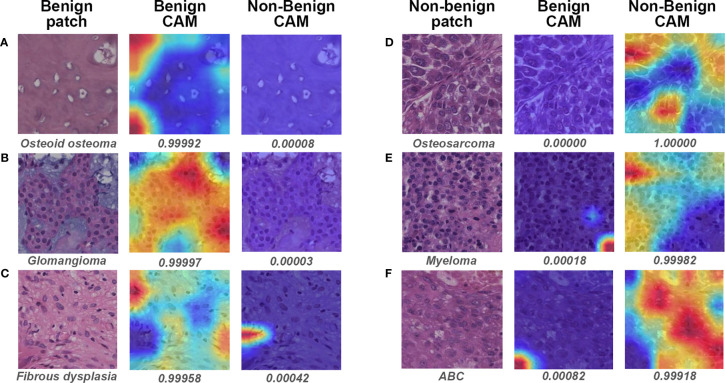
Gradient-weighted class activation mapping (Grad-CAM) in binary classification for representative patches of slides correctly classified by both models and pathologist #1. The specific classification is shown under the original patch, and the predictive probability for CAM of each class is shown below the corresponding Grad-CAM heatmap. **(A–C)** show the representative patches of benign bone tumors, whereas **(D–F)** show the representative patches of non-benign bone tumors. CAM, class activation mapping; ABC, aneurysmal bone cyst.

**Figure 6** illustrates the heatmaps of Grad-CAM results for binary classification. For most benign cases, the model identified the widespread stromal area without cells (**Figure 6A**) or stromal areas with scattered benign cells (**Figure 6C**) as essential regions for benign prediction. In some particular cases of benign tumors that share highly similar “dense-cell” microscopic morphology with non-benign tumors, the model effectively differentiated the confusing area as the benign region (**Figure 6B**). Visualization for non-benign patches showed that the different arrangements of atypical cells were deemed by the model as discriminative features for non-benign prediction (**Figures 6D–F**).

**Figure 7:**
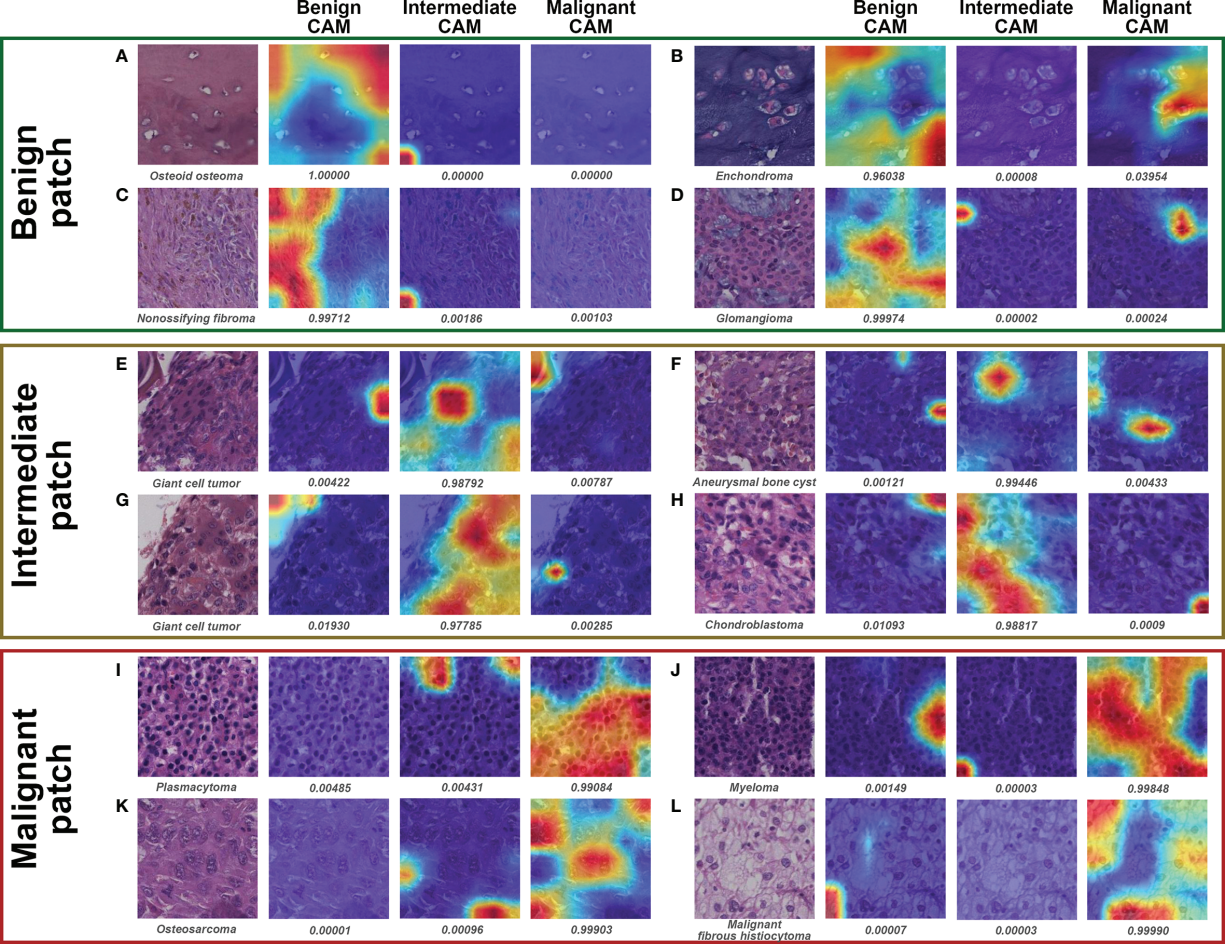
Grad-CAM in ternary classification for representative patches of slides correctly classified by both models and pathologist #1. The specific classification is shown under the original patch, and the predictive probability for CAM of each class is shown below the corresponding Grad-CAM heatmap. **(A–D)**, **(E–H)**, and **(I–L)** show the representative patches of benign, intermediate, and malignant bone tumors, respectively.

Visualization of representative patches for ternary classification is shown in **Figure 7**. The mechanism for benign prediction of ternary classification (**Figures 7A–D**) was similar to that of binary classification. Intriguingly, the model could accurately identify the specific structures, such as giant cells (**Figures 7E–G**) and chondroblasts (**Figure 7H**), as important regions for intermediate classification. Furthermore, the highly dense organization of atypical cells (**Figures 7I, J**) and the combination of stroma and scattered malignant cells (**Figures 7K, L**) were regarded by the model as morphological features for malignant prediction.

### 3.4 Case Review

**Figure 8:**
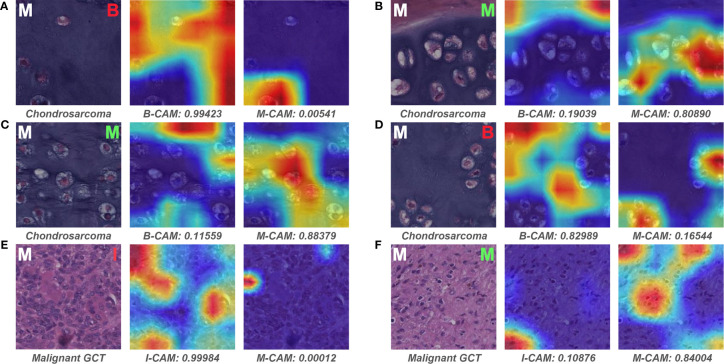
Representative patches of slides wrongly classified by models and the associated Grad-CAM results. The ground truth label of the original patch is displayed on the upper left in white, and the predictive classification of the model is presented on the upper right in red (false prediction) or green (correct prediction). The specific classification is shown under the original patch, and the predictive probability for CAM of each class is shown below the corresponding Grad-CAM heatmap. **(A–F)** show the representative patches of chondrosarcoma and malignant giant cell tumor, respectively. B, benign; I, intermediate; M, malignant; CAM, class activation mapping; GCT, giant cell tumor.

We examined the slides that pathologist #1 correctly predicted, whereas both models wrongly classified. Interestingly, all six malignant slides that were classified as benign by models belonged to chondrosarcoma. After visualizing some of the patches for these six slides, we found that the model could favorably recognize atypical cells for chondrosarcoma in the patch level (**Figures 8B, C**). However, there were numerous patches of the normal interterritorial matrix (**Figures 8A, D**), which were unintentionally cropped by pathologists as ROI, for one chondrosarcoma slide. This kind of patch-level annotation noise was remarkable in chondrosarcoma, causing the number of noise patches to overcome that of the true malignant patches in the averaging process of slide-level prediction. In addition, both models classified one malignant slide as intermediate, and this slide turned out to be a malignant giant cell tumor (GCT). The mechanism of such erroneous slide-level prediction was also associated with the annotation noise (similar to that of chondrosarcoma), although the patch-level discriminative features were successfully identified by the model (**Figure 8F**). This malignant GCT was mainly composed of the normal GCT area that was characteristic of giant cells (**Figure 8E**), whereas malignant cells only constituted a small part of the annotated ROI.

**Figure 9:**
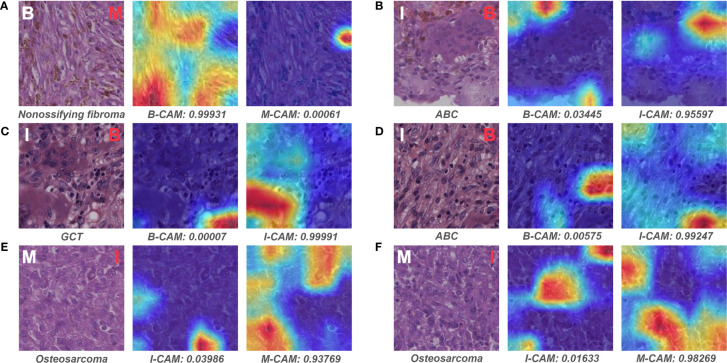
Representative patches of slides wrongly classified by pathologist #1 and the associated Grad-CAM results. The ground truth label of the original patch is displayed on the upper left in white, and the predictive classification of pathologist #1 is presented on the upper right in red (false prediction). The specific classification is shown under the original patch, and the predictive probability for CAM of each class is shown below the corresponding Grad-CAM heatmap. **(A–F)** show the representative patches of benign, intermediate, and malignant bone tumors, respectively. B, benign; I, intermediate; M, malignant; CAM, class activation mapping; ABC, aneurysmal bone cyst; GCT, giant cell tumor.

**Figure 9** depicts the representative patches of slides that pathologist #1 incorrectly classified but both models correctly predicted. For the benign bone tumor that has seemingly malignant microscopic structures, the model could effectively differentiate the associated patches as benign classification (**Figure 9A**). In addition, the model also showed favorable performance in identifying specific features of the intermediate slides that the pathologist was unsure of diagnosing solely based on the microscopic assessment (**Figures 9B–D**). Furthermore, for patches of malignant slides that share similar cell arrangements with intermediate cases, the model could easily and correctly distinguish the corresponding area with high predictive probability (**Figures 9E, F**).

## 4 Discussion

In this preliminary study, we found that several widely proved DL models trained with limited pathological slides could effectively classify bone tumors histopathologically in terms of aggressiveness. The VGG-16 and Inception V3, which defeated other models in patch-level performance, showed comparable diagnostic abilities with the senior pathologist and triumphed over attending and resident pathologists in slide-level predictive performance. Moreover, we discovered that the DL model could extract specific visual features of each classification and relied on them to make favorable predictions.

In the conventional clinical setting, a patient who is suspected of bone tumor usually undergoes clinical and radiological examinations for an initial assessment. However, many cases are challenging for physicians to give definitive or qualitative diagnoses solely based on patient history or plain radiographs. Therefore, a tissue biopsy is needed under such circumstances to determine the tumor’s biological nature, thus directing more appropriate treatment (5). The qualitative classification for bone tumor after biopsy is usually divided into benign, intermediate, and malignant tumor according to the aggressiveness evaluated under microscopy. Intermediate and malignant cases can be grouped as non-benign tumors because they normally require subsequent interventions. Patients diagnosed with benign bone tumors are generally requested for regular follow-up after one-stage resection during biopsy surgery. In comparison, secondary surgery that includes extensive resection and structural fixation is commonly required for intermediate bone tumors owing to the moderate recurrence rate and the local aggressiveness. For malignant bone tumors, extensive resection with implant support is combined with chemotherapy or radiotherapy for cases without metastasis, whereas palliative therapy is needed for metastatic cases (1). Given that it is difficult for general pathologists to accurately classify bone tumors histopathologically because of the low incidence and tumor heterogeneity, the expected goal of the current study was to build a DL-based classifier that reaches the diagnostic level of pathologists from the academic medical center.

The focus of AI-related research for bone tumor diagnosis is mainly on the radiographic analysis for the moment (27, 28). Bao et al. (29) have incorporated various features from radiographic observations and demographic information to build a naïve Bayesian-based model for ranking and classifying a wide range of bone tumor diagnoses. Yu et al. (30) have established a DL algorithm to classify bone tumors in terms of aggressiveness on plain radiographs, finding the model has the ROC curve AUC of 0.877 for binary classification (benign *vs*. non-benign) and the CKS of 0.560 for ternary classification on testing dataset. However, the radiological information is relatively limited for AI models to train and learn because only several radiographic images can be obtained from one patient diagnosed with the bone tumor. The bone tumor’s morphological information presented on the radiograph can be inconsistent due to the variabilities of radiation intensity, patient position, and film magnification. Therefore, DL models may not grasp sufficient discriminative features only from limited radiographs from one patient, whereas a much more sample size per class is needed to control the overfitting for a DL model with more than hundreds of thousands of parameters (31). In contrast, the current study used WSI to scan almost all cell-level image data from the pathological slide, which was then exported as hundreds of thousands of image patches for training the DL model. Six selected models showed satisfactory learning curves, but models’ performances in differentiating the bone tumor’s pathological data are different from that in distinguishing the ImageNet dataset, where the VGG-16 and Inception V3 showed better results in our dataset. The best models trained with more than 400,000 histopathological image patches in this study also showed relatively higher patient-level (slide-level) predictive capabilities in binary and ternary classification for bone tumors compared with that of models trained with limited radiological data (30), reaching the diagnostic level of senior pathologists while outperforming attending and junior pathologists.

The tumor area annotated by pathologists instead of the whole tissue area on the pathological slide was used as ROI for patch extraction in this study because of the following three reasons. First, the histopathological component of bone tumors is usually mixed with various normal connective tissues (bone, cartilage, vascular tissue, fibrous tissue, and muscular tissue), which would introduce a lot of noise data for DL models if the normal area is used as training input. Second, the atypical tumor areas that exclude normal connective tissue areas are relatively easy for general pathologists to identify. Third, given the huge morphological heterogeneity (various origins, such as osteogenic, chondrogenic, and fibrous) among bone tumors from each qualitative class and the moderate slide sample size of this study, it is impractical to automate the ROI selection process using algorithms for the moment. Some slides with suboptimal stain quality were excluded before WSI scanning in this study, making the pipeline workflow not clinically applicable enough. An updated algorithm including stain normalization and defective slide detection should be integrated into the model in the future.

The patch-level Grad-CAM visualization showed that the DL model could overcome the interference of histomorphological heterogeneity among the same class, well-differentiating bone tumors in terms of aggressiveness according to the diagnostic feature. We speculate that the under-differentiated and non-specific abstract features in non-benign bone tumors could be effectively extracted and learned by the DL model to make the correct patch-level binary prediction. However, for malignant bone tumors with atypical cell components accounting for a small proportion of the whole slide, the DL model gave wrong slide-level predictions because noise patches (patches of benign structure) unintentionally produced in the ROI selection process was used for slide-level probability calculation. Such label noise is inevitable when the pathologist annotates an ROI area, and many weakly supervised approaches have been attempted to address this issue and reduce annotation workload (10, 32). Therefore, given the histopathological diversity for bone tumors of the same qualitative classification, future studies with more sample sizes that have numerous cases of different origins in each class are needed to build an annotation-free DL classifier with high performance.

Most of the current DL-based histopathological diagnosis system has been built as the assistant role for human pathologists (11, 33) because of the related ethical issues of entirely relying on DL models (8). It is usually devastating for pathologists to miss the diagnosis of a non-benign bone tumor in adolescents that could have been properly managed. Therefore, a screening tool with high sensitivity would assist inexperienced pathologists in general hospitals to confidently exclude non-benign bone tumors and refer suspected aggressive cases to specialized hospitals for further treatment. The best-performing DL model in this study showed a comparable sensitivity and a higher specificity compared with the senior pathologist in slide-level prediction, indicating the promising value of DL in screening non-benign bone tumors in the future. Besides histopathological features, pathologists typically use radiological and demographic findings as references to reach the final clinical diagnosis of the bone tumor. However, when given the pathological information alone, the evaluation results among pathologists seemed not consistent in this study, which shows that the human’s classification of bone tumors may be unreliable solely based on the histopathological assessment. Later DL-related research should focus on combining clinical, radiological, and histopathological data of bone tumors, along with cutting-edge approaches like the ensemble model (34), to raise the sensitivity to near 100% while maintaining the high specificity of the model.

To our knowledge, this is the first study that verifies the feasibility of using the DL-based model to classify bone tumors histopathologically in terms of aggressiveness. In contrast, previous related works only concentrated on the histologic analysis of specific diagnoses of bone tumors and had small numbers of WSI slides (35–37). Considering the low prevalence of bone tumors and the relative difficulty to obtain biopsy tissues compared with radiographs, the sample size of more than 700,000 patches generated from 427 slides was fairly adequate to train a DL model. With the help of the Grad-CAM, we found that the model could easily differentiate some cases that were confusing for pathologists. The visualization also helped us partly interpret the DL underlying mechanism of classifying bone tumors, which was deemed a black box that was hard to explain before. These results would provide a theoretical basis for the future application of DL-assisted histopathological diagnosis for bone tumors.

There exist several limitations in our study. First, this is a single-center study with a moderate number of pathological slides. The variety in the process of slide preparation and WSI scanning from different institutions may have an impact on the image quality of training input. Therefore, the model’s generalizability might be partially limited by the training dataset of this study, and a multi-center research should assist in achieving a more robust result in the future. Second, the label noise (wrongly labeled patches) generated from manually annotated ROIs would introduce information bias to some degree for DL training, although such bias could be mostly compensated in the averaging process of the slide-level prediction. Third, due to the retrospective nature of the data acquisition, the number of slides in each classification was not well balanced, thus bringing in selection bias for training and evaluation of the model. However, we used the average metric weighted by each class to minimize this kind of bias. Moreover, the subjectivity of pathologists in determining tumor areas would also result in selection bias, and future studies are needed to address this problem with weakly supervised or unsupervised DL models. Fourth, there were few specific cases with the rare incidence in each qualitative classification (for example, fibrous histiocytoma and fibrosarcoma). The DL model may not be trained well to extract and learn morphological features specific to these rare cases based on the limited number of representative patches. Future studies should include more data about rare cases to make the model more generalizable. Fifth, there exist some non-neoplastic lesions mimicking bone tumors radiographically or histopathologically, such as osteomyelitis and osteonecrosis. Our DL models were only trained on the neoplastic lesions, leading to inapplicability to differentiate such non-neoplastic lesions, although these kinds of tumor mimics are relatively easy for pathologists to distinguish from neoplastic lesions based on the laboratory test and the histological absence of neoplastic cells. Finally, histopathological results of bone tumors are more often combined with clinical and radiological features of patients for pathologists to predict the clinical classification, whereas we only focused on the histopathological side in this study. In order to make the model’s underlying prediction mechanism closer to the human being, later research should consider integrating multiple levels of data to train a comprehensive DL model.

In summary, the present study shows that the DL model can effectively classify primary bone tumors histopathologically in terms of aggressiveness, reaching the predictive performance similar to the senior pathologist while higher than attending and resident pathologists. These results are promising and would help expedite the future application of DL-assisted histopathological diagnosis for primary bone tumors.

## Data Availability Statement

The original data presented in the study are included in the article/**Supplementary Material**. Further inquiries can be directed to the corresponding authors.

## Ethics Statement

The studies involving human participants were reviewed and approved by the Ethics Committee of the First Affiliated Hospital of Chongqing Medical University. Written informed consent to participate in this study was provided by the participants’ legal guardian/next of kin.

## Author Contributions

YuT, XH, and JZ conceived the study. YuT, XH, CL, and JZ contributed to developing the methodology. YuT, HW, WJ, YCh, WY, MG, BT, and MY performed the data collection. CL developed the algorithms for deep learning training. CL, ZL, and KG provided the experimental and computational resources. YiT, CW, JL, and YCa performed the histopathological evaluation. YuT, XH, and CL analyzed the data. TC, AZ, YCa, and JZ supervised the execution of the study. YuT wrote the original draft. CL, JZ, and TC edited and revised the manuscript. All authors contributed to the article and approved the submitted version.

## Funding

This study was supported by the Chongqing Science and Health Joint Medical Research Project from the Chongqing Municipal Health Commission [No. 2020MSXM002]; the Chongqing Postgraduate Research and Innovation Project from the Chongqing Municipal Education Commission [No. CYB20142]; and the General Project of Technology Innovation and Application Development from the Chongqing Science and Technology Commission [No. cstc2019jscx-msxm0156]. The funders had no role in study design, data collection and analysis, decision to publish, or preparation of the manuscript.

## Conflict of Interest

Authors ZL and KG are employed by Chongqing Defang Information Technology Co., Ltd.

The remaining authors declare that the research was conducted in the absence of any commercial or financial relationships that could be construed as a potential conflict of interest.

## Publisher’s Note

All claims expressed in this article are solely those of the authors and do not necessarily represent those of their affiliated organizations, or those of the publisher, the editors and the reviewers. Any product that may be evaluated in this article, or claim that may be made by its manufacturer, is not guaranteed or endorsed by the publisher.

## References

[1] Santini-Araujo E Kalil RK Bertoni F Park Y-K. Tumors and Tumor-Like Lesions of Bone. London, UK: Springer Nature (2020).

[2] Siegel RL Miller KD Fuchs HE Jemal A. Cancer Statistics, 2021. CA Cancer J Clin (2021) 71:7–33. doi: 10.3322/caac.21654 33433946

[3] Mangham DC Athanasou NA. Guidelines for Histopathological Specimen Examination and Diagnostic Reporting of Primary Bone Tumours. Clin Sarcoma Res (2011) 1:1–13. doi: 10.1186/2045-3329-1-6 22613930 PMC3351796

[4] Fletcher CDM Bridge JA Hogendoorn PCW Mertens F World Health Organization International Agency for Research on Cancer. WHO Classif Tumours Soft Tissue Bone. Lyon: IARC Press (2013).

[5] Franchi A. Epidemiology and Classification of Bone Tumors. Clin Cases Miner Bone Metab (2012) 9:92–5. PMC3476517 23087718

[6] Salto-Tellez M Maxwell P Hamilton P. Artificial Intelligence-the Third Revolution in Pathology. Histopathology (2019) 74:372–6. doi: 10.1111/his.13760 30270453

[7] Albawi S Mohammed TA Al-Zawi S. Understanding of a Convolutional Neural Network. In: 2017 International Conference on Engineering and Technology (ICET). IEEE (2017). p. 1–6.

[8] Niazi MKK Parwani AV Gurcan MN. Digital Pathology and Artificial Intelligence. Lancet Oncol (2019) 20:e253–61. doi: 10.1016/S1470-2045(19)30154-8 PMC8711251 31044723

[9] Duggento A Conti A Mauriello A Guerrisi M Toschi N. Deep Computational Pathology in Breast Cancer. Semin Cancer Biol (2020) 72:226–37. doi: 10.1016/j.semcancer.2020.08.006 32818626

[10] Chen CL Chen CC Yu WH Chen SH Chang YC Hsu TI. An Annotation-Free Whole-Slide Training Approach to Pathological Classification of Lung Cancer Types Using Deep Learning. Nat Commun (2021) 12:1193. doi: 10.1038/s41467-021-21467-y 33608558 PMC7896045

[11] Song Z Zou S Zhou W Huang Y Shao L Yuan J. Clinically Applicable Histopathological Diagnosis System for Gastric Cancer Detection Using Deep Learning. Nat Commun (2020) 11:4294. doi: 10.1038/s41467-020-18147-8 32855423 PMC7453200

[12] Kott O Linsley D Amin A Karagounis A Jeffers C Golijanin D. Development of a Deep Learning Algorithm for the Histopathologic Diagnosis and Gleason Grading of Prostate Cancer Biopsies: A Pilot Study. Eur Urol Focus (2021) 7:347–51. doi: 10.1016/j.euf.2019.11.003 PMC7242119 31767543

[13] Chuang WY Chang SH Yu WH Yang CK Yeh CJ Ueng SH. Successful Identification of Nasopharyngeal Carcinoma in Nasopharyngeal Biopsies Using Deep Learning. Cancers (Basel) (2020) 12:507. doi: 10.3390/cancers12020507 PMC7072217 32098314

[14] Bankhead P Loughrey MB Fernández JA Dombrowski Y McArt DG Dunne PD. QuPath: Open Source Software for Digital Pathology Image Analysis. Sci Rep (2017) 7:1–7. doi: 10.1038/s41598-017-17204-5 29203879 PMC5715110

[15] Hou L Samaras D Kurc TM Gao Y Davis JE Saltz JH. Patch-Based Convolutional Neural Network for Whole Slide Tissue Image Classification. In: Proceedings of the IEEE Conference on Computer Vision and Pattern Recognition. IEEE (2016). p. 2424–33. 10.1109/CVPR.2016.266 PMC5085270 27795661

[16] Dimitriou N Arandjelović O Caie PD. Deep Learning for Whole Slide Image Analysis: An Overview. Front Med (2019) 6:264. doi: 10.3389/fmed.2019.00264 PMC6882930 31824952

[17] Krizhevsky A Sutskever I Hinton GE. Imagenet Classification With Deep Convolutional Neural Networks. Adv Neural Inf Process Syst (2012) 25:1097–105. doi: 10.1145/3065386

[18] Simonyan K Zisserman A. Very Deep Convolutional Networks for Large-Scale Image Recognition. arXiv [Preprint] (2014). Available at: https://arxiv.org/abs/1409.1556

[19] Szegedy C Vanhoucke V Ioffe S Shlens J Wojna Z. Rethinking the Inception Architecture for Computer Vision. In: Proceedings of the IEEE Conference on Computer Vision and Pattern Recognition. IEEE (2016). p. 2818–26.

[20] Huang G Liu Z van der Maaten L Weinberger KQ. Densely Connected Convolutional Networks. In: Proceedings of the IEEE Conference on Computer Vision and Pattern Recognition. IEEE (2017). p. 4700–8.

[21] He K Zhang X Ren S Sun J. Deep Residual Learning for Image Recognition. In: Proceedings of the IEEE Conference on Computer Vision and Pattern Recognition. IEEE (2016). p. 770–8.

[22] Tan M Chen B Pang R Vasudevan V Sandler M Howard A. Mnasnet: Platform-Aware Neural Architecture Search for Mobile. In: Proceedings of the IEEE/CVF Conference on Computer Vision and Pattern Recognition. IEEE (2019). p. 2820–8.

[23] Smith LN. Cyclical Learning Rates for Training Neural Networks. In: 2017 IEEE Winter Conference on Applications of Computer Vision (WACV). IEEE (2017). p. 464–72.

[24] Selvaraju RR Cogswell M Das A Vedantam R Parikh D Batra D. Grad-Cam: Visual Explanations From Deep Networks *via* Gradient-Based Localization. In: Proceedings of the IEEE International Conference on Computer Vision. IEEE (2017). p. 618–26.

[25] DeLong ER DeLong DM Clarke-Pearson DL. Comparing the Areas Under Two or More Correlated Receiver Operating Characteristic Curves: A Nonparametric Approach. Biometrics (1988) 44:837–45. doi: 10.2307/2531595 3203132

[26] Vanbelle S Albert A. A Bootstrap Method for Comparing Correlated Kappa Coefficients. J Stat Comput Simul (2008) 78:1009–15. doi: 10.1080/00949650701410249

[27] Li MD Ahmed SR Choy E Lozano-Calderon SA Kalpathy-Cramer J Chang CY. Artificial Intelligence Applied to Musculoskeletal Oncology: A Systematic Review. Skeletal Radiol (2021). doi: 10.1007/s00256-021-03820-w 34013447

[28] Vogrin M Trojner T Kelc R. Artificial Intelligence in Musculoskeletal Oncological Radiology. Radiol Oncol (2020) 55:1–6. doi: 10.2478/raon-2020-0068 33885240 PMC7877260

[29] Do BH Langlotz C Beaulieu CF. Bone Tumor Diagnosis Using a Naïve Bayesian Model of Demographic and Radiographic Features. J Digit Imaging (2017) 30:640–7. doi: 10.1007/s10278-017-0001-7 PMC5603428 28752323

[30] He Y Pan I Bao B Halsey K Chang M Liu H. Deep Learning-Based Classification of Primary Bone Tumors on Radiographs: A Preliminary Study. EBioMedicine (2020) 62:103121. doi: 10.1016/j.ebiom.2020.103121 33232868 PMC7689511

[31] Benkendorf DJ Hawkins CP. Effects of Sample Size and Network Depth on a Deep Learning Approach to Species Distribution Modeling. Ecol Inform (2020) 60:101137. doi: 10.1016/j.ecoinf.2020.101137

[32] Campanella G Hanna MG Geneslaw L Miraflor A Werneck Krauss Silva V Busam KJ. Clinical-Grade Computational Pathology Using Weakly Supervised Deep Learning on Whole Slide Images. Nat Med (2019) 25:1301–9. doi: 10.1038/s41591-019-0508-1 PMC7418463 31308507

[33] Wang S Yang DM Rong R Zhan X Fujimoto J Liu H. Artificial Intelligence in Lung Cancer Pathology Image Analysis. Cancers (Basel) (2019) 11:1–16. doi: 10.3390/cancers11111673 PMC6895901 31661863

[34] Salvi M Molinari F Iussich S Muscatello LV Pazzini L Benali S. Histopathological Classification of Canine Cutaneous Round Cell Tumors Using Deep Learning: A Multi-Center Study. Front Vet Sci (2021) 8:640944. doi: 10.3389/fvets.2021.640944 33869320 PMC8044886

[35] Mishra R Daescu O Leavey P Rakheja D Sengupta A. Convolutional Neural Network for Histopathological Analysis of Osteosarcoma. J Comput Biol (2018) 25:313–25. doi: 10.1089/cmb.2017.0153 29083930

[36] Arunachalam HB Mishra R Daescu O Cederberg K Rakheja D Sengupta A. Viable and Necrotic Tumor Assessment From Whole Slide Images of Osteosarcoma Using Machine-Learning and Deep-Learning Models. PloS One (2019) 14:1–19. doi: 10.1371/journal.pone.0210706 PMC6469748 30995247

[37] Fu Y Xue P Ji H Cui W Dong E. Deep Model With Siamese Network for Viable and Necrotic Tumor Regions Assessment in Osteosarcoma. Med Phys (2020) 47:4895–905. doi: 10.1002/mp.14397 32677073

