# Supplementary material for: Qualitative Histopathological Classification of Primary Bone Tumors Using Deep Learning: A Pilot Study
Source: Front Oncol. 2021 Oct 6;11:735739. doi: 10.3389/fonc.2021.735739 (PMC8526973; doi:10.3389/fonc.2021.735739)
Supplement: Supplementary file 1 [file DataSheet_1.pdf]

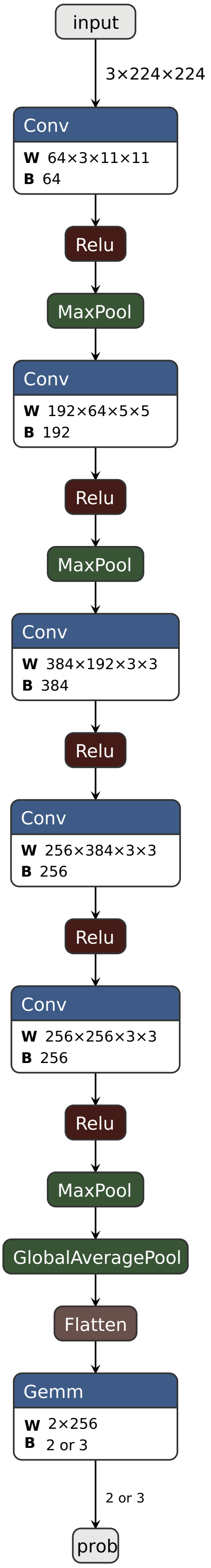

Supplementary Figure S1. AlexNet architecture. Number of parameters for binary and ternary classification: 2,470,210 & 2,470,467. The second and third fully connected layers from the last in the original network version was removed to avoid overfitting because the classification task is simple in this study and then replaced by global average pooling for better visualization. Conv, Convolution; W, Weight; B, Bias; Relu, Rectified Linear Unit; MaxPool, Max pooling; GlobalAveragePool, Global average pooling; Gemm, General matrix multiplication; prob, probability; Mul, Multiplication; Add, Addition; Concat, Concatenate; AveragePool, Average pooling; BatchNormilization, Batch mormalization; var, variance.

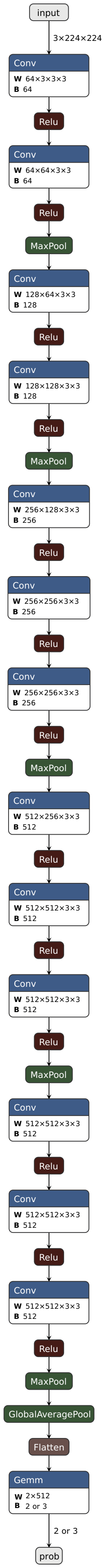

Supplementary Figure S2. VGG-16 architecture. Number of parameters for binary and ternary classification: 14,724,162 & 14,724,675. The second and third fully connected layers from the last in the original network version was removed to avoid overfitting because the classification task is simple in this study and then replaced by global average pooling for better visualization. Abbreviation is shown in the legend of figure S1. Zoom out for the detailed information.

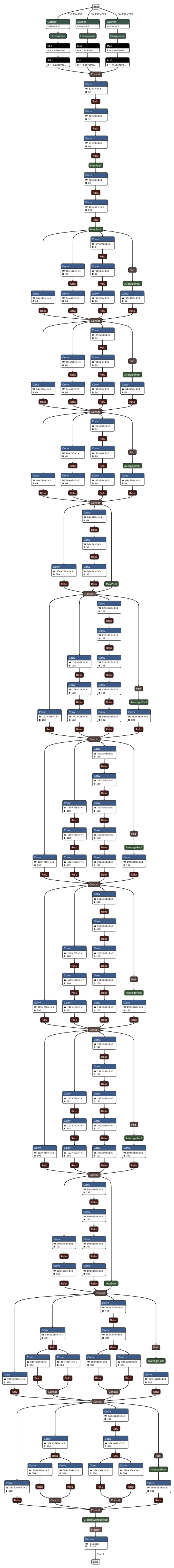

Supplementary Figure S3. Inception V3 architecture. Number of parameters for binary and ternary classification: 24,348,900 & 24,351,718. Abbreviation is shown in the legend of figure S1. Zoom out for the detailed information.

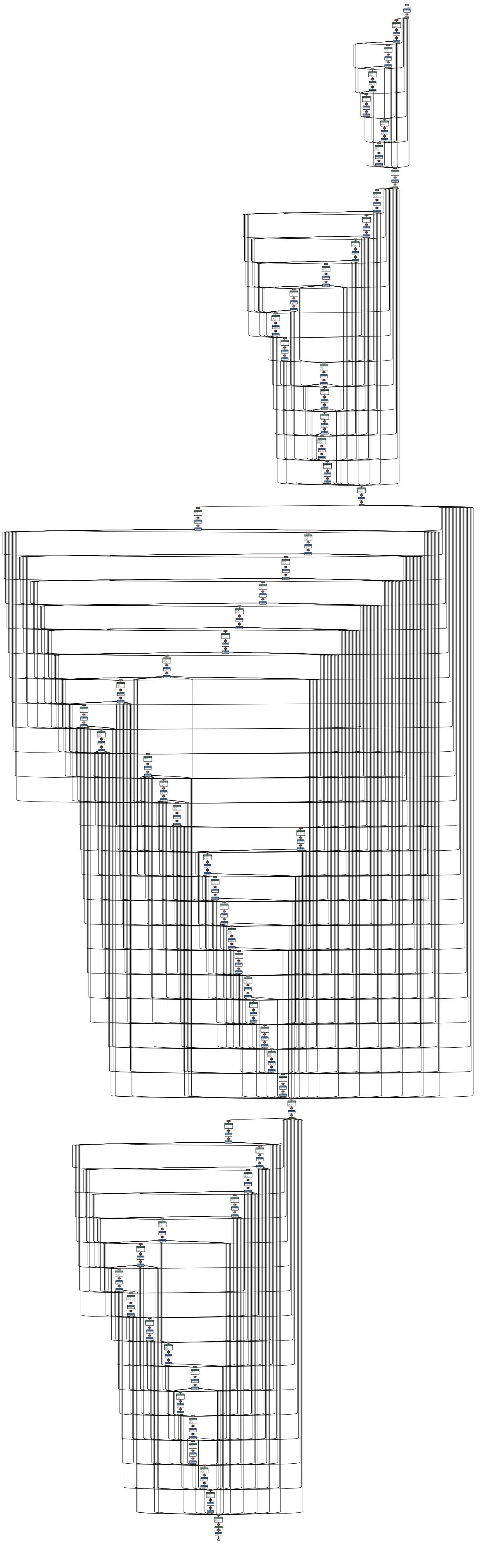

Supplementary Figure S4. DenseNet-121 architecture. Number of parameters for binary and ternary classification: 6,955,906 & 6,956,931. Abbreviation is shown in the legend of figure S1. Zoom out for the detailed information.

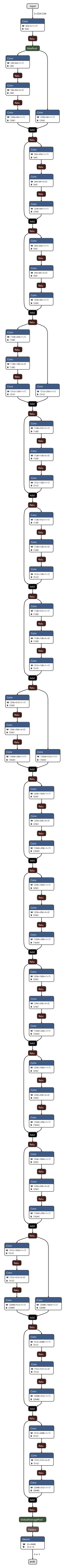

Supplementary Figure S5. ResNet-50 architecture. Number of parameters for binary and ternary classification: 23,512,130 & 23,514,179. Abbreviation is shown in the legend of figure S1. Zoom out for the detailed information.

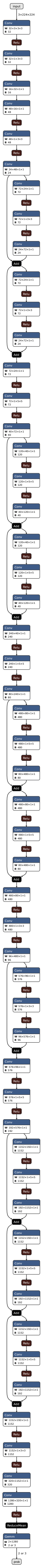

Supplementary Figure S6. MnasNet architecture. Number of parameters for binary and ternary classification: 3,104,874 & 3,106,155. Abbreviation is shown in the legend of figure S1. Zoom out for the detailed information.

Supplementary Table S1. Information of definitive diagnosis for the included cases in each group

Aggressiveness	Definitive diagnosis	Number of cases
**Benign**	Osteochondroma	56
	Enchondroma	29
	Hemangioma	23
	Osteoid osteoma	18
	Glomangioma	16
	Nonossifying fibroma	14
	Fibrous dysplasia	12
	Chondroma	9
	Osteofibrous dysplasia	8
	Fibrous histiocytoma	8
	Osteoma	5
**Intermediate**	Giant cell tumor	40
	Aneurysmal bone cyst	26
	Chondroblastoma	12
	Desmoplastic fibroma	5
	Osteoblastoma	3
**Malignant**	Osteosarcoma	57
	Chondrosarcoma	27
	Plasmacytoma	20
	Myeloma	10
	Chordoma	8
	Lymphoma of bone	5
	Undifferentiated sarcoma	4
	Malignant giant cell tumor	3
	Malignant fibrous histiocytoma	3
	Ewing sarcoma	3
	Angiosarcoma	1
	Fibrosarcoma of bone	1
	Epithelioid Hemangioendothelioma	1

Supplementary Table S2. Slide information

	Slide number	Training (70%)	Validation (15%)	Testing (15%)
**Benign**	198	138	30	30
**Intermediate**	86	60	13	13
**Malignant**	143	99	22	22
**Total**	427	297	65	65

Supplementary Table S3. Image patch information

	Patch number	Training (70%)	Validation (15%)	Testing (15%)
**Benign**	262,515	164,886	50,273	47,356
**Intermedi**	136,023	104,103	17,548	14,372
**Malignant**	318,300	203,631	54,901	59,768
**Total**	716,838	472,620	122,722	121,496

Supplementary Table S4. Patch-level ternary classification report of different models

	B-Pre	B-Rec	B-F1	I-Pre	I-Rec	I-F1	M-Pre	M-Rec	M-F1
**AlexNet**	0.73	0.86	0.79	0.41	0.74	0.53	0.80	0.54	0.65
**VGG-16**	0.77	0.89	0.83	0.49	0.86	0.62	0.88	0.61	0.72
**Inception v3**	0.75	0.92	0.82	0.49	0.83	0.62	0.89	0.58	0.70
**ResNet-50**	0.73	0.87	0.79	0.45	0.80	0.58	0.82	0.54	0.65
**DenseNet-121**	0.78	0.88	0.83	0.44	0.88	0.59	0.86	0.57	0.68
**MnasNet**	0.75	0.86	0.80	0.50	0.83	0.63	0.84	0.61	0.71

B, benign; I, intermediate; M, malignant; Pre, precision; Rec, recall; F1, F1-score.

Supplementary Table S5. Slide-level ternary classification report of models and pathologists

	B-Pre	B-Rec	B-F1	I-Pre	I-Rec	I-F1	M-Pre	M-Rec	M-F1
**VGG-16**	0.82	0.90	0.86	0.87	1.00	0.93	0.82	0.64	0.72
**Inception v3**	0.81	1.00	0.90	0.93	1.00	0.96	1.00	0.64	0.78
**Pathologist #1**	0.88	0.77	0.82	0.75	0.69	0.72	0.74	0.91	0.82
**Pathologist #2**	0.76	0.93	0.84	0.50	0.31	0.38	0.75	0.68	0.71
**Pathologist #3**	0.68	0.90	0.77	0.60	0.46	0.52	0.80	0.55	0.65
**Pathologist #4**	0.65	0.93	0.77	0.50	0.31	0.38	1.00	0.64	0.78

B, benign; I, intermediate; M, malignant; Pre, precision; Rec, recall; F1, F1-score.
